# Serum Amyloid A3 Gene Expression in Adipocytes is an Indicator of the Interaction with Macrophages

**DOI:** 10.1038/srep38697

**Published:** 2016-12-08

**Authors:** Yohei Sanada, Takafumi Yamamoto, Rika Satake, Akiko Yamashita, Sumire Kanai, Norihisa Kato, Fons AJ van de Loo, Fusanori Nishimura, Philipp E. Scherer, Noriyuki Yanaka

**Affiliations:** 1 Graduate School of Biosphere Science, Hiroshima University, Higashi-Hiroshima, 739-8528, Japan; 2 Kyushu University Faculty of Dental Science, Fukuoka, Japan; 3 Experimental Rheumatology, Department of Rheumatology, Radboud University Medical Center, Nijmegen, The Netherlands; 4 Touchstone Diabetes Center, Department of Internal Medicine, University of Texas Southwestern Medical Center, USA

## Abstract

The infiltration of macrophages into adipose tissue and their interaction with adipocytes are essential for the chronic low-grade inflammation of obese adipose tissue. In this study, we identified the *serum amyloid A3 (Saa3*) gene as a key adipocyte-derived factor that is affected by interaction with macrophages. We showed that the Saa3 promoter in adipocytes actually responds to activated macrophages in a co-culture system. Decreasing C/EBPβ abundance in 3T3-L1 adipocytes or point mutation of C/EBPβ elements suppressed the increased promoter activity in response to activated macrophages, suggesting an essential role of C/EBPβ in Saa3 promoter activation. Bioluminescence based on Saa3 promoter activity in Saa3-luc mice was promoted in obese adipose tissue, showing that Saa3 promoter activity is most likely related to macrophage infiltration. This study suggests that the level of expression of the *Saa3* gene could be utilized for the number of infiltrated macrophages in obese adipose tissue.

Obesity, in particular excess visceral adiposity, and obesity-related metabolic disorders have emerged as crucial health issues worldwide. These metabolic disorders are collectively referred to as metabolic syndrome and increase the risk of developing type 2 diabetes, cardiovascular disease, and cancers^1^,^2^. Overnutrition, involving excess nutrient intake that exceeds energy expenditure, and a sedentary lifestyle contribute to the aberrant accumulation of white adipose tissue through adipocyte hyperplasia and hypertrophy, leading to pathological changes of adipose functions^1^,^2^. Previous reports have clearly showed that expanding adipose tissue is well characterized as a chronic state of low-grade inflammation that is strongly linked to alterations of cellular composition and the abnormal production of cytokines, including tumor necrosis factor α (TNF-α), interleukin (IL)-1, and monocyte chemoattractant protein-1 (MCP-1/CCL2)^2^,^3^,^4^,^5^,^6^.

More recent research has indicated that obesity induces the accumulation of immune cells, including macrophages, T cells, and B cells, in adipose tissue^4^,^5^,^6^,^7^,^8^,^9^,^10^,^11^. In particular, macrophage infiltration into adipose tissue is an essential event for fat inflammation and systemic metabolic disorders because the number of macrophages in adipose tissue correlates with the degree of obesity, and is linked to adipose inflammation and insulin resistance^4^,^5^,^7^,^11^. In fact, a lack of MCP-1/CCL2 or its receptor, CC motif chemokine receptor-2 (CCR-2), was shown to reduce macrophage accumulation in obese adipose tissue and fat inflammation, and to improve systemic insulin sensitivity in high-fat diet (HFD) fed mice compared with wild-type mice^12^,^13^. Moreover, weight loss induced by surgery or exercise and conditional ablation of macrophages using the CD11c promoter resulted in a reduction in the number of adipose tissue macrophages of obese mice in parallel to the decreased production of pro-inflammatory cytokines and suppressed systemic insulin resistance^14^,^15^,^16^. In particular, recent works have strongly suggested that crosstalk between adipocytes and infiltrated macrophages plays a crucial role in chronic adipose inflammation and adipose remodeling^10^,^17^,^18^,^19^,^20^,^21^,^22^. Hence, determination of the number of infiltrated macrophages and *in vivo* evaluation of their interaction with adipocytes are essential for assessment of the chronic inflammatory state in obese adipose tissue.

Our recent study showed that, in mice supplemented with an anti-inflammatory vitamin, vitamin B6, macrophage marker gene expression and macrophage infiltration in obese adipose tissue were specifically down-regulated^20^. Furthermore, we performed a comparative analysis on the gene expression of obese adipose tissue of *db*/*db* mice and with vitamin B6 supplementation. Using this approach and by obtaining transcriptomic data, candidate genes related to the increase in infiltrated macrophages in adipose tissue *in vivo* were identified^20^,^22^. In this study, because activated macrophages could spread systemically in obese mice, we focused on adipocyte-derived genes that are differentially regulated by interaction with activated macrophages in order to establish a new non-invasive *in vivo* model for evaluation of the adipose inflammatory state associated with macrophage infiltration. We isolated the adipocyte-derived *serum amyloid A3 (Saa3)* gene for the monitoring of macrophage infiltration in adipose tissue. We characterized the mouse Saa3 promoter region upstream of the luciferase (Saa3-luc) reporter chimeric gene in order to monitor macrophage infiltration. Finally, we generated Saa3-luc transgenic mice (Saa3-luc mice) and subjected them to *in vivo* luminescence imaging analysis upon being fed an HFD. *In vivo* assessment of the chronic low-grade inflammatory state of obese fat by determining macrophage infiltration would be important to evaluate the preventive effect of pharmaceutical agents and functional food against obesity-related disorders.

## Results

### *Saa3* gene expression in adipocytes is up-regulated by interaction with infiltrated macrophages

**Figure 1:**
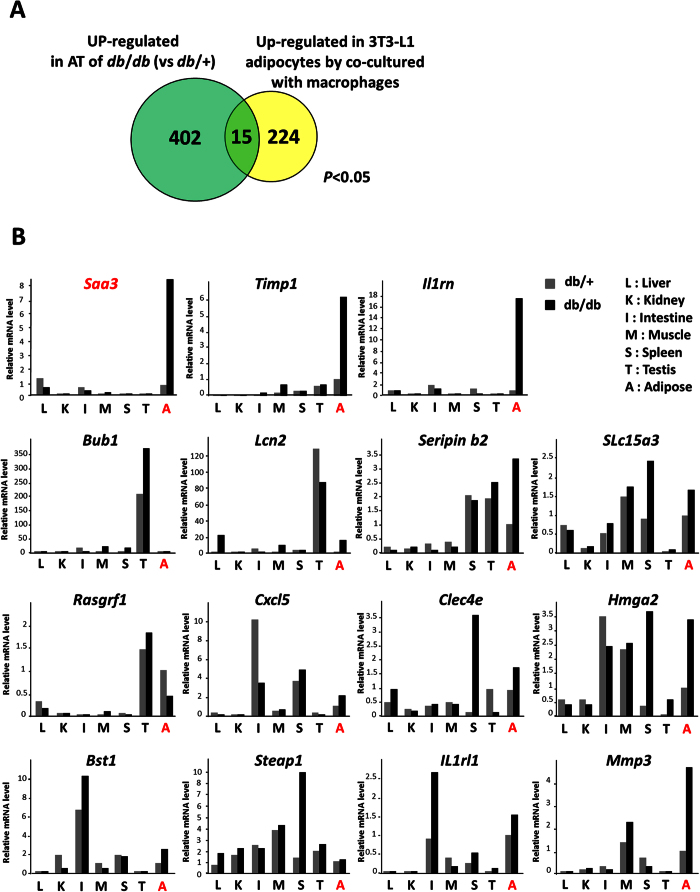
Analysis of two transcriptomes isolates Saa3 gene whose expression is upregulated by the interaction with macrophages. (**A**) The venn diagram shows genes that are upregulated in the db/db white adipose tissue and up-regulated in 3T3-L1 adipocytes by the interaction with activated RAW264.7 macrophages. Of a total 402 genes up-regulated in db/db adipose tissue, the expression of 15 genes was also increased in 3T3-L1 adipocytes by the interaction with activated macrophages *(p* < 0.05). (**B**) Total RNAs from several types of tissue of db/+ and db/db mice were isolated. The relative mRNA expression level of each gene was determined by quantitative PCR and normalized to L19 mRNA level. The data are representative of two independent experiments.

**Figure 2:**
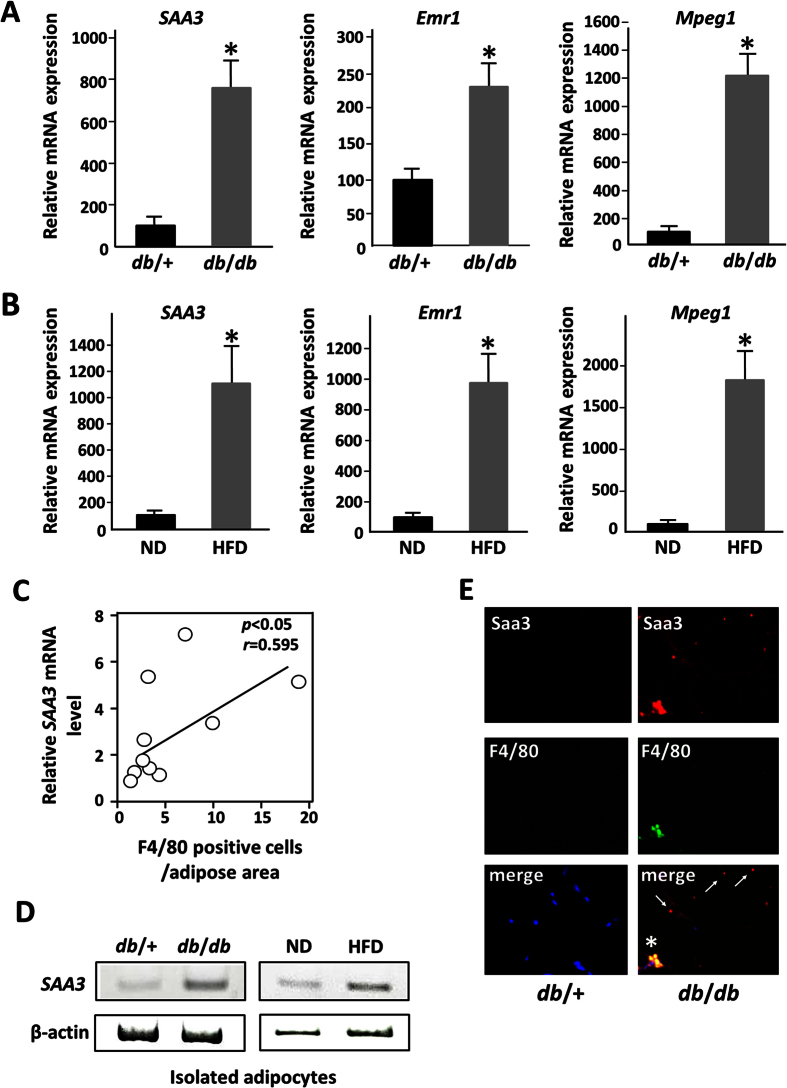
Saa3 gene expression in adipocytes is up-regulated by the interaction with macrophages. (**A**) Total RNAs from individual mice (*n* = 3) were subjected to quantitative PCR. Values are normalized to L19 mRNA level. *p < 0.05 compared with that of control mice (*db/+*). The data (mean ± S.E.) are representative of two independent experiments. (**B**) Mice were divided into two groups (*n* = 4), and fed basal diet (*ND*) or a high-fat diet (*HFD*) for 16 weeks. The relative mRNA expression level of each gene was determined by quantitative PCR and normalized to L19 mRNA level. *p < 0.05 compared with that of mice with normal diet (*ND*). The data (mean ± S.E.) are representative of two independent experiments. (**C**) The relative mRNA expression level of Saa3 gene in adipose tissue of mice fed HFD (*n* = 12) was determined by quantitative PCR and normalized to β-actin mRNA level. Pearson’s correlation coefficient showed a positive correlation between Saa3 mRNA level and number of *F4/80* positive cells in adipose tissue of mice fed HFD. (**D**) Mature adipocytes were isolated from white adipose tissue of db/+, db/db, ND and HFD mice as described under “Materials and Methods”. Semiquantitative RT-PCR was performed to determine mRNA levels of *Saa3* and β-actin. (**E**) An immunohistochemical study was carried out using 7-μm-thick paraffin-embedded sections of the epididymal adipose tissue from db/db mice for the macrophage marker F4/80 (Serotec, Oxford, UK), followed by Alexa Fluor 488-labeled anti-rat IgG. The rabbit Saa3 antibody was visualized with Cy3-labeled anti-rabbit IgG. The nuclei were stained with 4′,6-diamidino-2-phenylindole (*blue*). Saa3 protein was co-localized with F4/80 (*) and also seen within vesicle-like structures in adipocytes (arrows).

To analyze the changes in gene expression in adipose tissue during the development of obesity, this study identified a gene cluster including 402 genes whose expression was shown by DNA microarray analysis to be fivefold higher in the adipose tissue of *db*/*db* mice than in that of *db*/+ mice. Moreover, we utilized an *in vitro* co-culture system using 3T3-L1 adipocytes and RAW264.7 macrophages and performed gene expression analysis on differentiated 3T3-L1 adipocytes cultured with stimulated or unstimulated RAW264.7 macrophages. We found that 224 adipocyte genes were significantly up-regulated more than fivefold upon co-culture with macrophages activated by lipopolysaccharide (LPS). Then, we carried out a comparative analysis based on these *in vivo* and *in vitro* transcriptomic data in order to identify *in vivo* adipocyte-derived genes that were possibly affected by infiltrated macrophages in obese adipose tissue (Fig. 1A). In this study, as shown in Table 1, we isolated 15 genes whose expression was significantly up-regulated more than fivefold in both *in vivo* and *in vitro* gene expression profiles. We further characterized the expression of these 15 genes and showed that the mRNA expression levels of three genes (*Saa3, Timp1*, and *Il1rn*) were specifically increased in obese adipose tissue of *db*/*db* mice (Fig. 1B). These observations indicated that these three genes can be considered to be key adipocyte-derived factors whose expression is up-regulated by interaction with macrophages and potentially available as indicators of the number of macrophages in obese adipose tissue. Furthermore, we selected the *Saa3* gene for the observation of macrophage infiltration into adipose tissue because *Saa3* gene expression in adipocytes was increased upon co-culture with macrophages with the strongest transcriptional induction (by 72-fold) among the three genes. As shown in Fig. 2A, *Saa3* gene expression in adipose tissue was significantly increased in *db*/*db* mice compared with that in *db*/+ mice as well as mRNA of macrophage marker genes such as *Emr1* and *Mpeg1*. We also performed quantitative RT-PCR analysis of the expression level of the *Saa3* gene in adipose tissue of mice with diet-induced obesity. In mice fed an HFD for 16 weeks, epididymal white fat mass was greater with the increased expression of the *Emr1* and *Mpeg1* mRNAs, whereas the *Saa3* mRNA level in adipose tissue was significantly up-regulated (11-fold) in obese mice with an HFD compared with that in non-obese mice (Fig. 2B). To determine whether *Saa3* expression levels in obese adipose tissue were correlated with macrophage infiltration, we measured *Saa3* and *Emr1* mRNA expression levels and F4/80-positive cell number in the epididymal white adipose tissue of individual mice fed an HFD and showed significant correlations between *Saa3* mRNA level and the number of *F4/80*-positive cells (*r* = 0.565, *p* < 0.05) (Fig. 2C) and *Emr1* mRNA level (*r* = 0.471, *p* < 0.05) (Supplementary Fig. 1A) by using Pearson’s correlation coefficient analyses. We further performed time course analyses of mRNA expression in adipose tissue of mice fed an HFD and observed that the upregulation of *Saa3* mRNA expression is paralleled by an increase in the *Emr1* mRNA expression (Supplementary Fig. 1B). Next, to determine the adipose tissue cell types that were responsible for these mRNA changes, we isolated mature adipocytes from epididymal adipose tissue by collagenase digestion. The expression of *Saa3* mRNA in the adipocyte fractions isolated from adipose tissue of *db*/*db* mice was up-regulated compared with that in *db*/+ mice (Fig. 2D). Similarly, we showed that *Saa3* mRNA expression increased in the adipocyte fraction isolated from mice fed an HFD (Fig. 2D). We further examined *Saa3* mRNA expression in adipocyte and macrophage cell lines and several types of cells in obese adipose tissue and compared mRNA expression profiles among the 15 candidate genes, suggesting that *Saa3* mRNA is expressed in both adipocytes and macrophages (Supplementary Fig. 2A). As shown in Supplementary Fig. 2B, we observed that *Saa3* mRNA is expressed in activated macrophage RAW264.7 cells and bone marrow-derived M1 macrophages. Further immunohistochemical analyses demonstrated that Saa3 protein is observed in F4/80-positive cells (macrophages) and is also seen within vesicle-like structures in adipocytes (Fig. 2E). Taken together, these observations suggest that *Saa3* gene expression in mature adipocytes responds to macrophages and that M1 macrophages were partially responsible for the *Saa3* mRNA in obese adipose tissue *in vivo*.

### Saa3 promoter activity is up-regulated in the presence of activated macrophages

**Figure 3:**
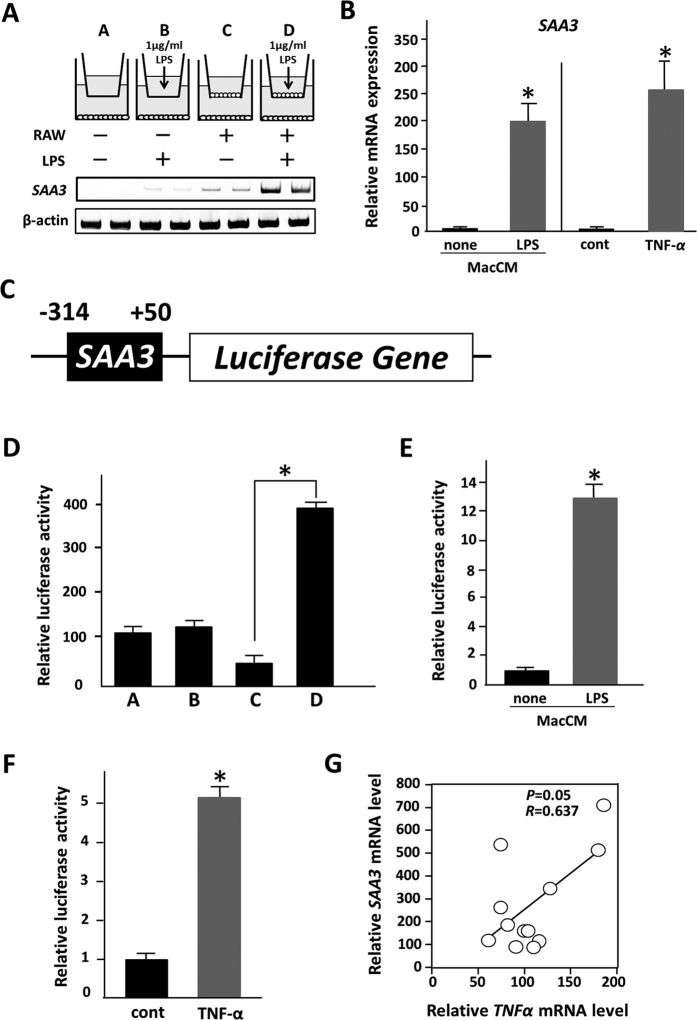
Saa3 mRNA expression in adipocytes is affected in the presence of macrophages. (**A**) 3T3-L1 preadipocytes were treated with MDI for 48 h and differentiated into mature adipocytes as described under “Materials and Methods”. RAW264.7 cells were stimulated with 1 μg/ml of LPS for 18 hr. Semiquantitative RT-PCR was performed to determine mRNA levels of *Saa3* and β-actin. (**B**) Differentiated 3T3-L1 adipocytes were treated with conditioned medium of RAW264.7 cells without LPS (*none*) or stimulated in the presence of 1 μg/ml of LPS for 18 hr (*LPS*). Differentiated 3T3-L1 adipocytes were treated with 10 ng/ml of TNF-α for 24 h. Total RNAs were extracted and subjected to quantitative PCR analysis to examine expression level of Saa3. The data (mean ± S.E.) are from a single experiment carried out (*n* = 3) and are representative of two independent experiments. All values are normalized to L19 levels. *p < 0.05. (**C**) The mouse Saa3 promoter region (−314/+50) was ligated upstream of the complete luciferase cDNA, generating an Saa3 promoter-luciferase chimeric gene (Saa3-luc). (**D**) 3T3-L1 adipocytes were infected with retroviruses harboring this chimeric gene (3T3-L1/Saa3-luc adipocytes). A co-culture system composed of 3T3-L1/Saa3-luc adipocytes and RAW264.7 macrophages were constructed (**A**). Luciferase activity in the 3T3-L1 adipocytes was examined upon co-culture with macrophages activated by LPS. *p < 0.05. (**E**) Differentiated 3T3-L1/Saa3-luc adipocytes were treated with conditioned medium of RAW264.7 cells without LPS (*none*) or stimulated in the presence of 1 μg/ml of LPS for 18 hr (*LPS*). (**F**) Differentiated 3T3-L1/Saa3-luc adipocytes were treated with 10 ng/ml of TNF-α for 24 h. Luciferase activity in the 3T3-L1 adipocytes was examined. The data (mean ± S.E.) are from a single experiment carried out (*n* = 3) and are representative of two independent experiments. *p < 0.05. (**G**) The relative mRNA expression level of Saa3 and TNF-α genes in adipose tissue of mice fed HFD (*n* = 12) was determined by quantitative PCR and normalized to β-actin mRNA level. Pearson’s correlation coefficient showed a positive correlation between Saa3 and TNF-α mRNA levels in adipose tissue of mice fed HFD.

The levels of *Saa3* mRNA in 3T3-L1 adipocytes were strikingly up-regulated upon co-culture with activated macrophages (Fig. 3A). The expression of *Saa3* mRNA in adipocytes was not affected by the presence of unstimulated macrophages or of LPS alone. Moreover, when differentiated 3T3-L1 adipocytes were treated with conditioned medium of macrophages activated by LPS (MacCM), *Saa3* mRNA expression level was significantly increased (Fig. 3B). Previous reports have shown that TNF-α secreted by activated macrophages is involved in the inflammatory response in obese adipose tissue, which is linked to obesity-related metabolic disorders including insulin resistance^11^,^17^. We showed here that exogenously added TNF-α increased *Saa3* gene expression in mature 3T3-L1 adipocytes (Fig. 3B), suggesting that TNF-α is one of potent inflammatory mediators secreted by activated macrophages to up-regulate *Saa3* mRNA expression in lipid-laden adipocytes.

Previously, Geurts *et al*. screened synovial genes whose expression is significantly up-regulated during the course of rheumatoid arthritis^23^. The Saa3 promoter (−314/+50) showed strong transcriptional induction in synovial fibroblasts in rheumatoid arthritis, suggesting that this promoter region is involved in the response to the inflammatory process in this disease^23^,^24^. Here, to examine whether the Saa3 promoter activity can respond to activated macrophages, we constructed an Saa3 promoter-luciferase chimeric gene (Saa3-luc) (Fig. 3C) and infected 3T3-L1 adipocytes with retroviruses harboring this gene (3T3-L1/Saa3-luc adipocytes). Next, we constructed a co-culture system composed of 3T3-L1/Saa3-luc adipocytes and RAW264.7 macrophages, and tested the Saa3 promoter activity in the adipocytes. Luciferase activity in the 3T3-L1 adipocytes was significantly elevated upon co-culture with macrophages activated by LPS treatment (Fig. 3D). In order to examine the effect of adherent RAW264.7 macrophages on the Saa3 promoter activity in adipocytes, we performed the co-culture of 3T3-L1 adipocytes and RAW264.7 cells in a contact system. RAW264.7 macrophages were directly plated onto 3T3-L1 adipocytes; the SAA3 promoter activity in adipocytes was examined (Supplementary Fig. 3), and was found to be up-regulated to the same extent as in the trans-well system, suggesting that the increase in the SAA3 promoter activity is mainly dependent on the secretion of factors such as TNF-α from macrophages. Moreover, we examined the effect of the medium conditioned with activated RAW264.7 macrophages on the Saa3 promoter activity in 3T3-L1 adipocytes. The addition of this conditioned medium markedly increased luciferase activity in 3T3-L1 cells (Fig. 3E). In this study, we further showed that the Saa3 promoter in mature 3T3-L1 adipocytes can respond to exogenously added TNF-α (Fig. 3F), suggesting that such promoter activity is partially up-regulated by TNF-α secreted by activated RAW264.7 macrophages. In addition, we analyzed *TNF-α* mRNA level in adipose tissue and tested its correlation with *Saa3* mRNA. In fact, there was significant correlation between *Saa3* mRNA level and *TNF-α* mRNA level (*r* = 0.637, *p* < 0.05) (Fig. 3G) by using Pearson’s correlation coefficient analysis. Although previous reports have shown that *Saa3* mRNA expression in adipocytes is up-regulated in response to saturated fatty acids^7^,^25^, the addition of palmitic acid conjugated with fatty acid-free BSA did not affect the Saa3 promoter activity (Supplementary Fig. 4).

In this study, we examined paracrine effects of secreted Saa3 on adipocytes. A previous work by Hiratsuka *et al*.^26^ demonstrated that Saa3 exerts its biological effects via its function as a ligand for the TLR4 receptor. We first analyzed the mRNA expression of the TLR4 receptor in 3T3-L1 cells and mature adipocytes derived from adipose tissues and RAW264.7 macrophages. The mRNA expression of TLR2 and TLR4 receptors in adipocytes was extremely low (Supplementary Fig. 5A), suggesting that Saa3 can not affect adipocyte functions. We further examined the biological effects of secreted Saa3 on adipocytes. Mouse Saa3 was overexpressed in HEK293 cells, and the condition medium was prepared from these HEK293 cells. This condition medium enhanced the phosphorylation of p65 (NF-κB) and extracellular signal-regulated kinase 1/2 proteins in RAW264.7 macrophages, which express TLR4 receptor; however, similar stimulation did not occur in differentiated adipocytes (Supplementary Fig. 5B).

### C/EBPβ is involved in the responsiveness of the Saa3 promoter to activated macrophages

**Figure 4:**
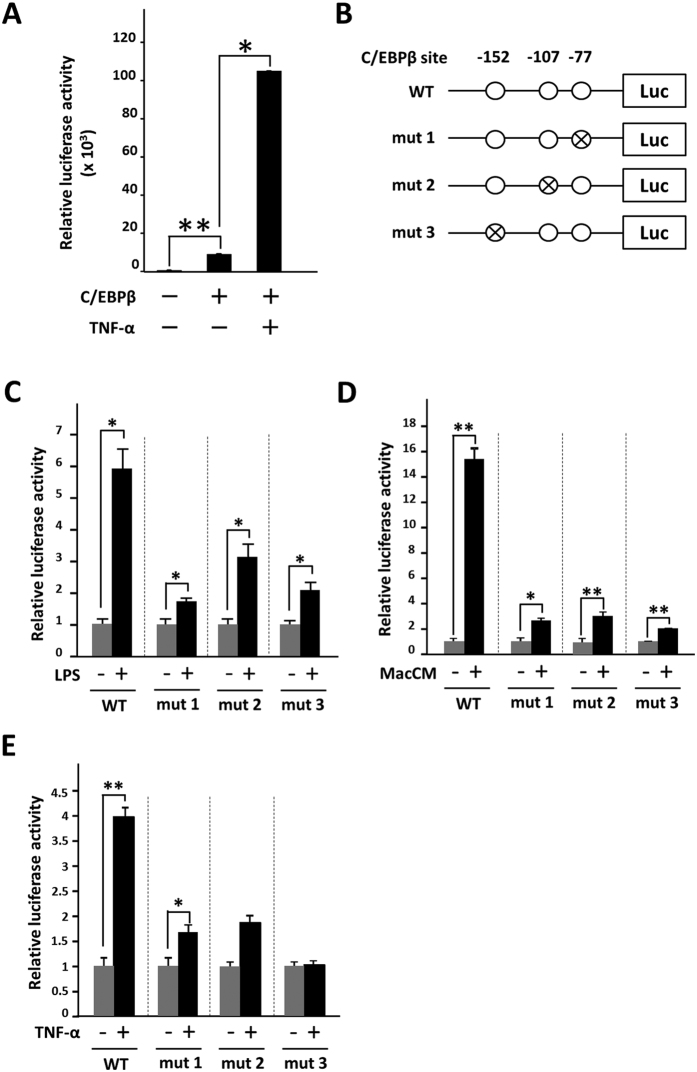
C/EBPβ is involved in Saa3 promoter activity in adipocytes in response to activated RAW264.7 macrophages. (**A**) HEK293T cells were transiently transfected with constructs for Saa3-luc with pcDNA-C/EBPβ or empty vector. After 2 days of the transfection, cells were treated with 10 ng/ml of TNF-α for 8 h, scraped and subjected to luciferase as described in Experimental Procedures. Data are means of triplicate experiments (mean ± S.E.). (**B**) Putative three C/EBPβ binding elements are located in the mouse Saa3 promoter region (−314/+50). Saa3 promoter was mutated and ligated upstream of the complete luciferase cDNA, generating mutant Saa3 promoter-luciferase chimeric genes (mut1, mut2 and mut3), respectively. (**C**) 3T3-L1 adipocytes were infected with retroviruses harboring each mutant promoter gene. A co-culture system composed of infected adipocytes and RAW264.7 macrophages were constructed. Luciferase activity in the 3T3-L1 adipocytes was examined upon co-culture with macrophages activated by LPS. *p < 0.05. (**D**) Infected 3T3-L1 adipocytes were treated with conditioned medium of RAW264.7 cells without LPS (*none*) or stimulated in the presence of 1 μg/ml of LPS for 18 hr (*LPS*). (**E**) Infected 3T3-L1 adipocytes were treated with 10 ng/ml of TNF-α for 24 h. Luciferase activity in the 3T3-L1 adipocytes was examined. The data (mean ± S.E.) are from a single experiment carried out (*n* = 3) and are representative of two independent experiments. *p < 0.05, ** *p* < 0.01.

**Figure 5:**
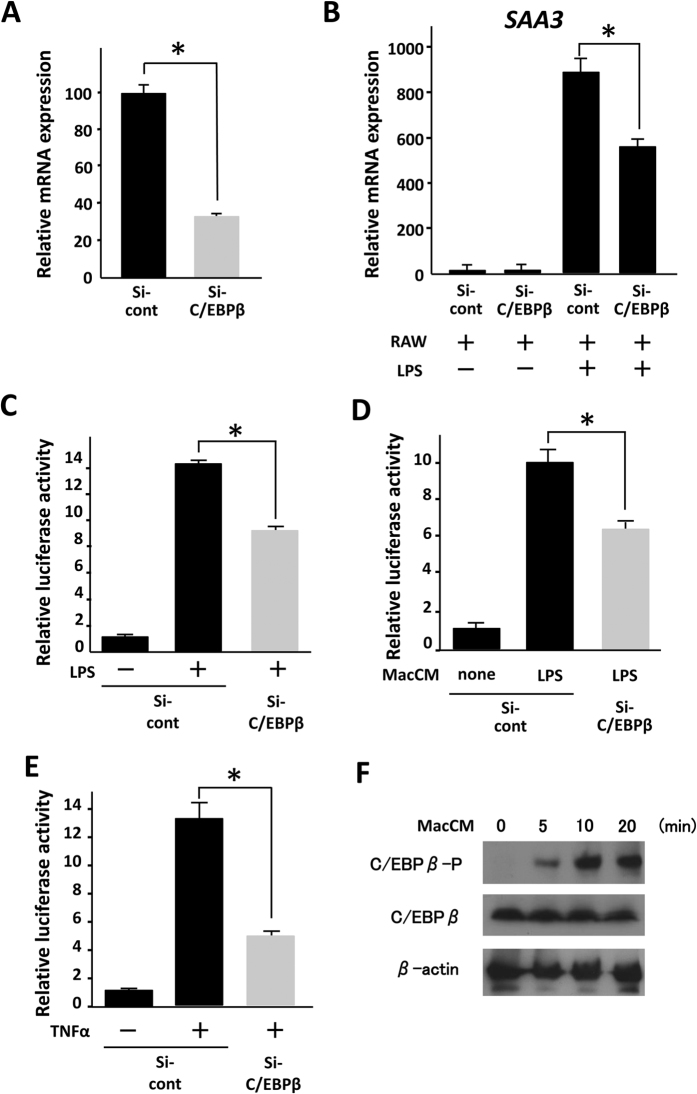
Effects of C/EBPβ siRNA on the Saa3 promoter activity in response to activated RAW264.7 macrophages. **(A)** 3T3-L1 preadipocytes were treated with MDI for 48 h and differentiated into mature adipocytes as described under “EXPERIMENTAL PROCEDURES”. Differentiated 3T3-L1 cells were transfected with control siRNA (si-cont) or C/EBPβ siRNA (si-C/EBPβ). After 2 days of transfection, total RNAs were extracted and subjected to quantitative PCR analysis (A) to examine the expression level of C/EBPβ mRNA. The level of L19 transcript was used as a control. (**B**,**C**) After 2 days of transfection, differentiated 3T3-L1 cells were subjected to the co-culture system with RAW264.7 cells. Saa3 mRNA level (**B**) and luciferase activity (**C**) in the 3T3-L1 adipocytes was examined upon co-culture with macrophages activated by LPS. *p < 0.05. (**D**) After 2 days of transfection, 3T3-L1 adipocytes were treated with conditioned medium of RAW264.7 cells without LPS (*none*) or stimulated in the presence of 1 μg/ml of LPS for 18 hr (*LPS*). (**E**) After 2 days of transfection, 3T3-L1 adipocytes were treated with 10 ng/ml of TNF-α for 24 h. Luciferase activity in the 3T3-L1 adipocytes was examined. (**F**) Mature 3T3-L1 cells were treated with MacCM for 5,10,15 minutes. p-CEBP/β, CEBP/β and β-actin proteins were detected by western blot analysis. The data (mean ± S.E.) are from a single experiment carried out (*n* = 3) and are representative of two independent experiments. *p < 0.05.

Three CCAAT/enhancer-binding protein β (C/EBPβ)-binding sites are present in the mouse Saa3 region (−152, −107, and −77)^24^,^27^. Because previous studies showed that C/EBPβ responds to inflammatory stimuli including IL-1 and TNF-α, we tested the role of C/EBPβ in the responsiveness of the Saa3 promoter to activated macrophages. In HEK293 cells, Saa3 promoter activity was dramatically up-regulated when C/EBPβ was overexpressed and enhanced by the addition of TNF-α (Fig. 4A). In this study, we generated three mutants of C/EBPβ-binding sites in the Saa3 promoter and infected 3T3-L1 adipocytes with retroviruses harboring these three mutant Saa3 promoters (mut1, mut2, and mut3) (Fig. 4B). We further tested the Saa3 promoter activity in the adipocytes using a co-culture system. Luciferase activity of wild-type Saa3 promoter in the 3T3-L1 adipocytes was significantly elevated upon co-culture with activated macrophages by nearly sixfold, whereas the promoter activities of the three mutants were up-regulated by 1.7- to 3.2-fold (Fig. 4C). Moreover, we examined the effect of MacCM and TNF-α on each of the Saa3 mutant promoters. Similarly, the three point mutations decreased the responsiveness to both MacCM and TNF-α (Fig. 4D,E). In particular, point mutation 3 (mut3) abolished the Saa3 promoter activation by TNF-α, suggesting the critical role of the C/EBPβ-binding site (−152) for the inflammatory response of the Saa3 promoter in adipocytes. To confirm the involvement of C/EBPβ in Saa3 promoter activation by the interaction with macrophages, we performed siRNA-mediated C/EBPβ knock-down in 3T3-L1 cells. The reduction in C/EBPβ mRNA level was assessed by quantitative RT-PCR (Fig. 5A). C/EBPβ silencing resulted in decreases in both *Saa3* mRNA and Saa3 promoter activity upon co-culture with macrophages activated by LPS (Fig. 5B,C). In addition, C/EBPβ RNAi gene silencing was shown to suppress the Saa3 promoter activation by MacCM and TNF-α (Fig. 5D,E). Moreover, we showed that phosphorylation of C/EBPβ on Thr188 in 3T3-L1 adipocyte was induced by MacCM addition (Fig. 5F). These findings strongly suggest that C/EBPβ actually regulates Saa3 promoter activity in the presence of activated macrophages.

**Figure 6:**
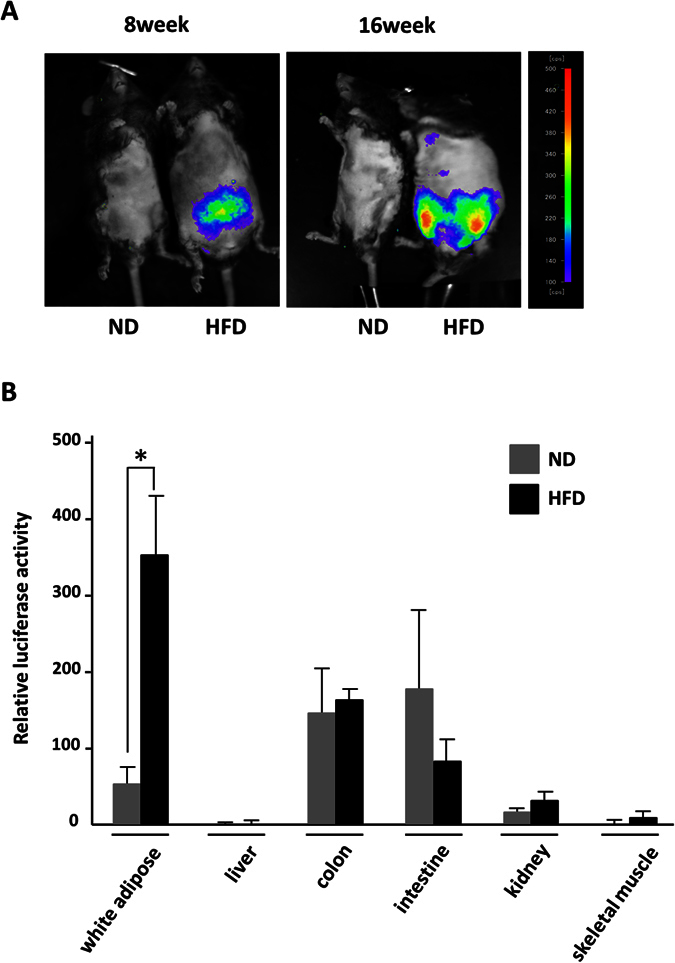
*In vivo* bioluminescent imaging analysis based on *Saa3* promoter activity in mice. (**A**) Transgenic mice carrying Saa3-luc were generated (Saa3-luc mouse). Saa3-luc mice were fed either ND or HFD for 8 weeks and 16 weeks, and subjected to *in vivo* bioluminescence analysis. (**B**) At the end of these 16 weeks, several types of tissue of Saa3-luc mice were isolated and subjected to the luciferase activity. The data (mean ± S.E.) are from a single experiment carried out (*n* = 3) and are representative of two independent experiments. *p < 0.05.

Finally, the Saa3 promoter-luciferase chimeric gene was applied to *in vivo* bioluminescent imaging analysis to monitor adipose inflammation caused by macrophage infiltration during the development of obesity. In this study, we generated Saa3-luc transgenic mice harboring Saa3-luc and two poly-A sites. F3 mice were divided into two groups and fed either HFD or normal diet (ND) for 16 weeks, and subjected to *in vivo* bioluminescence analysis. At the end of these 16 weeks, *in vivo* bioluminescent imaging revealed that obese adipose tissue of Saa3-luc mice fed HFD60 could be visualized by luciferase activity (Fig. 6A). We isolated several types of tissue from Saa3-luc mice and confirmed that the luciferase activity based on Saa3 promoter activity was significantly increased in obese adipose tissue compared with that in non-obese adipose tissue (Fig. 6B). Moreover, LPS administered by intraperitoneal injection did not show any bioluminescence in the SAA3-luc mice (data not shown).

## Discussion

The infiltration of macrophages into adipose tissue is associated with obesity; the crosstalk between adipocytes and infiltrated macrophages has been considered as an important pathological phenomenon during adipose tissue inflammation^10^,^11^,^12^,^13^,^14^,^15^,^16^. In order to evaluate the number of macrophages during adipose tissue inflammation induced by HFD, quantitative analysis of the mRNA of macrophage markers, such as *Emr1* and *Ccl2*, and immunohistological analysis of the proportion of adipose tissue sections stained positively for F4/80 are most commonly used. However, since obese adipose tissue is excised from obese mice for such analyses, a large number of mice are needed in these investigations and there has been limited opportunity to study the detailed pathological processes in each individual animal. Recently, *in vivo* imaging technologies have been developed by using a bioluminescence reporter system, and extensively applied for studies of molecular mechanisms in living organisms because the luciferase gene is not present in mammalian physiology^28^,^29^. To date, luminescence assays of luciferase reporter systems produced using a transgene or by viral infection have been utilized for the real-time biomedical monitoring of tumor development, inflammatory disease, circadian oscillations, and viral infection in living cells or whole animals^30^,^31^,^32^,^33^,^34^,^35^. In this study, we isolated the *Saa3* gene as an adipocyte-derived factor that is tightly associated with the increased infiltration of macrophages by a comparative analysis between *in vivo* and *in vitro* gene clusters. We have shown that the level of *Saa3* mRNA was positively correlated with the number of macrophages in adipose tissue *in vivo* and also that the level of *Saa3* mRNA was specifically up-regulated in adipose tissue of obese mice; then, we established transgenic mice carrying the *luciferase* gene under the control of the Saa3 promoter and subjected them to *in vivo* luminescence imaging analysis. Because the luciferase activity was detected in obese adipose tissue, it was shown that Saa3 promoter activity could be utilized for monitoring the adipose inflammatory state, possibly serving as an index of the number of infiltrated macrophages in adipose tissue for the following reasons. 1) *Saa3* mRNA expression is correlated with the number of macrophages (F4/80-positive cells) and with the mRNA expression of *Emr1*, which is a marker of macrophages in adipose tissue. 2) *Saa3* mRNA expression is down-regulated on administration of a diet rich in vitamin B6 (data not shown), which can decrease the number of macrophages in adipose tissue^20^. 3) The up-regulation of *Saa3* mRNA expression is paralleled by an increase in the *Emr1* mRNA expression in adipose tissue during obesity development. 4) A short-term HFD (for 1 week) possibly activated resident macrophages in adipose tissue, but, in this study, neither *Saa3* mRNA expression nor Saa3 promoter activity (luciferase activity) in adipose tissue was altered after a 1-week HFD (Supplementary Fig. 6). On the other hands, in previous work on a transcription factor, nuclear factor (NF)-κB, which is involved in numerous inflammatory responses, transgenic mice that carry the *luciferase* gene under the control of NF-κB were developed^31^,^35^. TNF-α and IL-1β, both of which can activate NF-κB signaling, are reportedly produced during adipose tissue inflammation, whereas recently obtained evidence has indicated that NF-κB activation plays an important role in the onset of insulin resistance^4^,^5^,^36^. Calsen *et al*. investigated NF-κB activation *in vivo* non-invasively in obese mice fed an HFD and showed that, interestingly, whole-body *in vivo* NF-κB activity increased more in the HFD group than in a control diet group^35^. In particular, in this previous study, the greatest effect of high-fat feeding was observed in the thoracic region, suggesting that these mice are widely available for the *in vivo* exploration of NF-κB activation; however, adipose tissue inflammation could not be visualized specifically by this bioluminescence imaging approach.

Here, we focused on the *Saa3* gene as an adipocyte-derived factor involved during adipose tissue inflammation, which sharply responds to these cells’ interaction with macrophages. The protein encoded by this gene is one of the members of the saa family, which are known as acute-phase proteins expressed in response to inflammation^37^, and has been studied in terms of the mRNA expression profile in chronic inflammatory diseases such as rheumatoid arthritis, atherosclerosis, and obesity^24^,^37^,^38^,^39^. The other members of Saa, Saa1 and Saa2, are primarily produced in the liver during acute inflammation^37^, whereas *Saa3* mRNA is highly expressed in extrahepatic tissue including lung, large intestine, and adipose tissue. Previous reports have shown that *Saa3* mRNA expression in adipocytes is increased in response to saturated fatty acids, IL-1β, and LPS *in vitro*^25^,^40^,^41^. In particular, *Saa3* expression in 3T3–L1 adipocytes is reportedly up-regulated in the presence of saturated fatty acid (palmitic acid) in a TLR4-dependent manner^7^,^25^. In this study, we examined whether the Saa3 promoter in mature 3T3-L1 adipocytes can respond to exogenously added palmitic acid, however, we could not observe any changes of Saa3 promoter activity. In addition, Saa3 protein which is also a ligand for TLR4 could not induce the phosphorylation of p65 (NF-κB) and ERK proteins in 3T3-L1 cells. These observations suggest that TLR4 expression in 3T3-L1 cells used in our experiments is quite low. However, because nutritional fatty acids, whose blood levels are often increased in obesity, have been shown to activate the TLR4 signaling and induce inflammatory signaling in adipocytes *in vivo*^7^, a possibility could not be excluded that saturated fatty acids were involved in the enhanced bioluminescence in the obese adipose tissue of mice with HFD. On the other hands, Van de Loo *et al*. previously explored targeted genes that are differentially regulated in the synovial membrane during the course of rheumatoid arthritis; they focused on the *Saa3* gene, whose transcriptional activity was shown to correlate most closely with disease severity^23^,^24^. Furthermore, the Saa3 promoter region (−314/+50) was shown to confer its cytokine-inducible expression in murine and human synovial fibroblasts, with the strongest transcriptional induction and strength. In this study, we showed that this promoter of the mouse Saa3 region (−314/+50) in adipocytes actually responds to activated macrophages in a co-culture system. Notably, an abundance of C/EBPβ-binding sites can be observed in the promoters of certain genes, including Saa3, whose expression correlates with the severity of collagen-induced arthritis^23^. Three C/EBPβ-binding sites are actually located in the mouse Saa3 region (−152, −107, and −77)^23^,^27^. Several studies have shown that C/EBPβ is transcriptionally activated by inflammatory stimuli, including inflammatory cytokines such as IL-6, IL-1, and TNF-α, and the role of C/EBPβ in the inflammatory cascade during the development of obesity has been discussed^42^,^43^,^44^. In this study, C/EBPβ actually functioned in Saa3 promoter activation by the interaction with macrophages and exogenously added TNF-α. This study also showed that C/EBPβ can be post-translationally activated via the phosphorylation of Thr-188. C/EBPβ is a well-studied key transcription factor that is up-regulated in the early stages of adipocyte differentiation. Previous reports have showed that the phosphorylation of Thr-188 in C/EBPβ by extracellular signal-regulated kinase induces subsequent phosphorylation on Ser-184 and Thr-179, resulting in DNA-binding function acquisition and transactivation^45^. In this study, macrophage-conditioned medium induced the phosphorylation of Thr-188, suggesting a potential role of macrophages in the modulation of adipogenesis. However, TNFα secreted from macrophages is well-known to strongly inhibit adipogenesis by downregulating PPARγ expression via NF-κB signaling^46^, which suggests that activated macrophages are involved in the inhibition of adipogenesis. Further studies are needed to reveal the molecular mechanism of C/EBPβ phosphorylation in adipocytes by the interaction with macrophages. A previous report showed that C/EBPβ deletion in obese *db/db* mice reduced adiposity, hepatic steatosis, and diabetes^42^. Furthermore, Rahman *et al*. demonstrated that bone marrow transplantation from mice lacking C/EBPβ reduced the levels of inflammatory markers and macrophages in adipose tissue, and maintained insulin sensitivity upon feeding on an HFD, suggesting the inflammatory roles of macrophage C/EBPβ in adipose tissue^44^. Meanwhile, C/EBPβ RNAi gene silencing was shown to inhibit NF-κB DNA-binding activity induced by palmitic acid treatment in 3T3-L1 adipocytes, whereas adenoviral-mediated over-expression of C/EBPβ significantly increased NF-κB binding activities in 3T3-L1 adipocytes^44^. These observations suggest a functional relationship between C/EBPβ and the transactivation potential of NF-κB.

**Figure 7:**
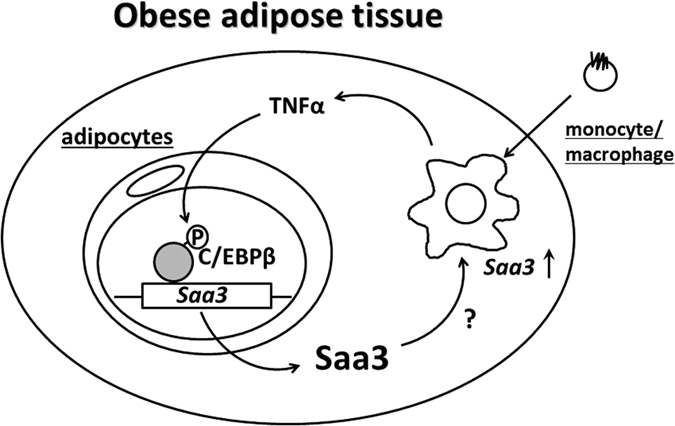
A model on Saa3 mRNA expression in a paracrine loop between adipocytes and activated macrophages. *Saa3* gene promoter in adipocytes responds to activated macrophages via C/EBPβ signaling. Saa3 mRNA expression could be utilized for monitoring the adipose inflammatory state, possibly serving as an index of the number of activated macrophages in obese adipose tissue.

Taking these findings together, the increase in luciferase activity in obese adipose tissue *in vivo* might be mediated by locally produced TNF-α, and the determination of Saa3 promoter activity would be useful for monitoring the adipose inflammatory state associated with increased macrophage content in adipose tissue (Fig. 7). As mentioned above, this study suggested that *Saa3* mRNA is also expressed in M1 macrophages in obese adipose tissue. Previous studies showed that HFD also increases the number of M1 macrophages in a variety of tissues such as livers^47^, pancreas^48^,^49^, skeletal muscles^50^,^51^, whereas, in this study, the luciferase activity was specifically increased in obese adipose tissue of SAA3-luciferase mice. Taken together, these observations suggest the enhanced bioluminescence in the obese adipose tissue is mainly dependent on an increased luciferase activity in the adipocytes, however, a possibility could not be excluded that Saa3 expression in M1 macrophages partially contributed to the enhanced bioluminescence in the obese adipose tissue in mice fed with HFD. On the other hand, a recent study revealed that Saa3 is an endogenous ligand of TLR4^26^ as mentioned above, so it started to be discussed in terms of having an inflammatory role via activation of the TLR4 signaling pathway. Previous reports have demonstrated that Saa3 induced in pre-metastatic lungs by S100A8 and S100A9 has a critical role in the accumulation of myeloid cells as a positive-feedback regulator for chemoattractant secretion in a TLR4-dependent manner^26^. Furthermore, a recent report has shown that Saa3 activates the NLRP3 inflammasome and promotes Th17 allergic asthma in mice^52^. Thus, because mouse Saa3 reportedly plays important roles during the pathological process of various diseases, these findings suggest that the mice established in this study would be widely available for the *in vivo* non-invasive exploration of compounds or food factors targeting *Saa3* gene expression.

## Methods

### Animals and diets

Male *db/db* (BKS.Cg-m^+/+^ Lepr db/J, 6 weeks old) and *db/+* (BKS.Cg-Dock7m^+/+^ Lepr db/J, 6 weeks old) mice were obtained from Charles River Japan. *db/db* mice fed high-fat diet (HFD: 60% of total calories from fat) and *db/+* mice fed AIN93G diet for 3weeks, respectively. Male C57BL/6j mice were purchased from Charles River Japan and they were divided into two groups. Seven-week old mice fed AIN93G (normal diet, ND) or HFD for 16 weeks. Male CD-1 (ICR) mice (4 weeks old, Charles River Japan) were housed in groups of 2 or 3 in metal cages. All mice were given free access to stock diet and deionized water, and housed in a room with controlled temperature (24 ± 1 °C) and a 12 h light/dark cycle; light from 0800 to 2000, daily. The animal study was approved by the Hiroshima University Animal Committee (Permit Number: C13-3), and the mice were maintained in accordance with the Hiroshima University Guidelines for the Care and Use of Laboratory Animals.

### siRNA, plasmid transfection and Cell culture

Mouse 3T3-L1 preadipocytes, mouse macrophage RAW264.7 cells and human HEK293 cells were cultured in a maintenance medium (10% fetal bovine serum, 100 units/ml penicillin and 100 μg/ml streptomycin in Dulbecco’s modified medium) at 37° in 5% CO_2_/95% humidified air. 3T3-L1 cells were differentiated as previously described^20^,^22^. Stimulation of RAW264.7 cells and co-cultivation of 3T3-L1 cells and RAW264.7 cells were performed based on previous protocol^20^,^22^. Differentiated 3T3-L1 adipocytes were stimulated with 10 ng/ml of mouse recombinant tumor necrosis factor-α (R&D system, Minneapolis, MN) for 24 hr. cDNA was amplified using a PCR primer set (5′-CCGCGTTCATGCACCGCCTG-3′ and 5′-ACCCGCGCCGCGCTAGCAGT-3′) designed according to the nucleotide sequences of mouse C/EBPβ and subcloned into pcDNA3.1, which generated pcDNA-C/EBPβ. DNA transfections were performed using GeneJuice Transfection Reagent (Merck Millipore) according to the manufacturer’s instructions. Small interfering RNA (siRNA) duplex oligoribonucleotides against mouse C/EBPβ were synthesized by Sigma. The sequences were as follows: sense 5′-CGGGUUUCGGGACUUGAUGTT-3′, antisense 5′-CAUCAAGUCCCGAAACCCGTT-3′.

Universal negative control siRNA (Sigma) was used as a control siRNA. Differentiated 3T3-L1 cells were transfected with these siRNAs to a final concentration of 20 nM using LipofectAMINE RNAimax (Invitrogen).

### DNA microarray analyses

Total RNAs were isolated from epididymal white adipose tissue of male *db*/*db* mice and *db*/+ mice using RNeasy lipid tissue kit (Qiagen Sciences, Germantown, MD), and pooled RNAs were subjected to cRNA synthesis for a DNA microarray analysis (44 K whole mouse genome 60-mer oligo microarray, Agilent Technologies, Palo Alto, CA). All procedures of fluorescence labeling, hybridization, and image processing were performed according to the manufacturer’s instructions. Gene expression data were obtained and statistically analyzed using Agilent Feature Extraction software (version 9.5). 3T3-L1 cells and RAW264.7 cells were co-cultured in maintenance medium using transwell system as described above and RAW264.7 cells were further stimulated with 1 μg/ml LPS for 24 hr. Total RNAs were isolated from 3T3-L1 cells using RNeasy kit (Qiagen). DNA microarray analysis, based on a system containing 5693 gene probes (Affymetrix, Santa Clara, CA, USA), was used to compare transcriptional profiles between 3T3-L1 cells co-cultured with RAW264.7 cells in the presence or absence of LPS. This array contains a broad range of genes derived from publicly available, well-annotated mRNA sequences. Preparations were quantified and their purity was confirmed by standard spectrophotometric methods. The results were expressed as the ratio of fluorescent intensity of the genes expressed in the two groups. The microarray data are also deposited in the NCBI GEO data base (available on the World Wide Web at www.ncbi.nlm.nih.gov/geo) under accession number GSE70527.

### Isolation of mature adipocytes

Epididymal white adipose tissue isolated from male *db/db, db/+,* ND and HFD mice were minced in phosphate-buffered saline and digested with 1 mg/ml collagenase Type I (Worthington Chemical Corporation) for 30 min at 37 °C. The resulting cell suspension was filtered through a 100-μm filter and centrifuged at 233 × *g* for 1 min to separate mature adipocytes from stromal vascular fraction (SVF) cells.

### RT-PCR Analyses

The reverse transcriptase reaction was carried out with 1 μg total RNA as a template to synthesize cDNA using ReverTra Ace (TOYOBO, Osaka, Japan) and random hexamers (TaKaRa Bio, Kyoto, Japan), according to the manufacturer’s instructions. For semi-quantitative PCR analysis, cDNA and primers were added to the GoTaq Master Mix (Promega, Madison, WI, USA) to give a total reaction volume of 20 μl. The reactions were sampled after 28 and 30 cycles under different PCR conditions, to monitor product accumulation. For quantitative PCR analysis, cDNA and primers were added to the THUNDERBIRD SYBR qPCR Mix (TOYOBO), to give a total reaction volume of 20 μl. PCR reactions were then performed using StepOnePlus^TM^ (Applied Biosystems, Foster City, CA). Conditions were set to the following parameters: 2 min at 95 °C, followed by 40 cycles each of 15 s at 95 °C and 1 min at 60 °C. The primers used for PCR analyses were as follows: Saa3, F, 5′-TTGATCCTGGGAGTTGACAG-3′, and R, 5′-CACTCATTGGCAAACTGGTC-3′; TNF-α, F, 5′-CCGATGGGTTGTACCTTGTC-3′, and R, 5′-CGGACTCCGCAAAGTCTAAG-3′; Emr1, F, 5′-ATTGTGGAAGCATCCGAGAC-3′, and R, 5′-GTAGGAATCCCGCAATGATG-3′; Mpeg1, F, 5′-GCTTGCCTCTGCATTTCTTC’, and R, 5′-TCTTCTGCTCCAGGTTTTGG-3′; L19, F, 5′-GGCATAGGGAAGAGGAAGG-3′, and R, 5′-GGATGTGCTCCATGAGGATGC-3′; C/EBPβ, F, 5′-ACAAGCTGAGCGACGGTAC-3′, and R, 5′-ACAGCTGCTCCACCTTCTTC-3′; β-actin, F, 5′-TTGGGTATGGAATCCTGTGGCATC-3′, and R, 5′-CGGACTCATCGTACTCCTGCTTGC-3′.

### Immunohistochemical analysis

The epididymal adipose tissue was isolated and fixed with neutral buffered formalin and embedded in paraffin. An immunohistochemical study was carried out using 4-μm-thick paraffin-embedded sections and rat anti-mouse F4/80 antibody (AbD Serotec, Raleigh, NC). The number of F4/80-positive cells in more than 100 serial fields was counted in a blinded fashion through the microscope, and the data were obtained as the mean number/mm^2^.

### Construction of Saa3-luc chimeric gene, retroviral transfection, and generation of chimeric mice

Restriction endonucleases and DNA-modifying enzymes were from TaKaRa Bio. The mouse Saa3 promoter region (−314/+50) was ligated upstream of the complete luciferase cDNA in pGEM3 (Promega), generating pSaa3-Luc. For retroviral expression, pSaa3-Luc was digested with both *Eco*RI and *Not*I, and the resulting DNA fragment containing the mouse Saa3 promoter region (−314/+50) and luciferase cDNA (*Saa3-Luc*) was ligated into *Eco*RI and *Not*I sites of pMX-puro retrovirus vector. High titer retroviruses harboring *Saa3-Luc* were produced in Phoenix 293 cells, and used to infect 3T3-L1 cells. After infection into 3T3-L1 cells, these cells were treated with 1 μg/ml puromycin for 7 days, and further co-cultured with RAW264.7 cells using a transwell system. Site-directed mutagenesis was performed using PrimeSTAR HS DNA Polymerase (TaKaRa Bio). The mutation was confirmed by DNA sequencing analysis. The primers used for the mutagenesis were as follows: mut 1, forward, 5′-TTATGCTTGATCAAACAGGGATTGCT-3′, and reverse, 5′-TTTGATCAAGCATAATCCCACTTACCC-3′; mut 2, forward, 5′-TTCTGATTGAGAAATTATGGGTAAGT-3′, and reverse, 5′-ATTTCTCAATCAGAAGATAACTTTTCC-3′; mut 3, forward, 5′-TGGCGCTTTCTGGGGAAAGAAGATGT-3′, and reverse, 5′-CCCCAGAAAGCGCCATCTAGGCATTTC 3′.

The transgene construct carrying *Saa3-Luc* and two polyadenylation sites was excised from agarose gel, purified, and used for microinjection into BDF1 mouse eggs at Japan SLC Inc. (Hamamatsu, Japan). Among 89 mouse pups, Tg10-3 line was studied. The male chimera harboring the Saa3-luc transgene was mated with C57BL/6 J female mice to obtain F1 offspring. The heterozygous F1 male offspring from this breeding were then backcrossed with purebred C57BL/6 J females to obtain F2 offspring; this process was continued until the F3 generation of mice was obtained. After weaning, the male heterozygous Saa3-luc transgenic mice were divided into two groups and fed HFD and AIN93G, respectively.

### Luciferase assay

For *in vitro* reporter studies, transfected 3T3-L1 (3T3-L1/Saa3-luc) cells were seeded at 1 × 10^5^ cells per ml in 12 well culture plate. Confluent 3T3-L1/Saa3-luc cells were differentiated as described above. Using a transwell system, 5 × 10^4^ RAW264.7 cells were cultured in the upper chamber and stimulated by LPS as mentioned above. 3T3-L1/Saa3-luc cells were stimulated with MacCM or 10 ng/ml of TNF-α for 24 hr. Subsequently, 3T3-L1/Saa3-luc cells were lysed in ice-cold lysis buffer (1% Triton-X 100, 2 mM DTT, 10% glycerol, and 25 mM Tris-HCl pH 7.5). After sonication, cell lysate was centrifuged at 12,000 rpm and supernatant was collected. Several tissues obtained from Saa3-luc mice were homogenized in the lysis buffer and centrifuged at 12,000 rpm. Luciferase activity was quantified using the luciferase assay kit (Toyo Inki, Japan) and a luminometer (Turner Model TD-20), expressed as relative light units normalized to total protein concentration of the cell or tissue extracts.

### Western blot analysis.

Mature 3T3-L1 adipocytes were stimulated by MacCM for 5, 10, 15 minutes. After stimulation, 3T3-L1 cells were washed with ice-cold PBS and scraped with RIPA buffer. Cells lysates were centrifuged at 12,000 rpm, 4 °C, 10 min for remove cell debris and supernatant were collected new tubes. Protein concentration of the supernatant was determined using the Bio-Rad protein assay kit (Bio-Rad) with BSA as a standard. 20 μg (protein equivalents) of the supernatant were resolved by SDS-PAGE, transferred onto a polyvinylidene difluoride (PVDF) membranes and immunoblotted with anti-CEBP/β (Santa Cruz Biotechnology), anti-p-CEBP/β (abcam) antibodies. Uncropped scans of the blots were supplied in Supplementary Fig. 7.

### *In vivo* bioluminescent imaging analysis

For *in vivo* bioluminescent imaging, male Saa3-luc mice were injected intraperitoneally with D-luciferin (150 mg/kg body weight, Promega) and 10 minutes later, anesthetized with pentobarbital sodium. After 5 minutes, Saa3-luc mice were placed supine position on the plate and imaged for 1 min with the camera set at the highest sensitivity by NightOWL II Imaging Systems LB983 (Berthold Technologies, Bad Wildbad, Germany). Photons emitted from tissues were analyzed using Indigo *in vivo* image software (Berthold). Signal intensity was quantified as the sum of all detected photon counts per second and presented as count/sec (cps). For any given analysis, all images were adjusted to the same scale of minimum and maximum luminescent intensity.

### Statistical analyses

Values are presented as means ± S.E. Statistical significance was determined by unpaired Student’s *t* test.

## Additional Information

**How to cite this article**: Sanada, Y. *et al*. Serum Amyloid A3 Gene Expression in Adipocytes is an Indicator of the Interaction with Macrophages. *Sci. Rep.* **6**, 38697; doi: 10.1038/srep38697 (2016).

**Publisher's note:** Springer Nature remains neutral with regard to jurisdictional claims in published maps and institutional affiliations.

## Supplementary Material

Supplementary Information

## Figures and Tables

**Table 1:** Analysis of two transcriptomes to isolate genes whose expression is upregulated by the interaction with macrophages.

GeneName	Accession No.	Description	Fold 1 (db/db)	Fold 2 (co-culture)
Hmga2	NM_010441	high mobility group AT-hook 2	38.0	5.0
Il1rn	NM_031167	interleukin 1 receptor antagonist, transcript variant 1	31.8	14.2
Cxcl5	NM_009141	chemokine (C-X-C motif) ligand 5	12.1	22.2
Bub1	NM_009772	budding uninhibited by benzimidazoles 1 homolog (S. cerevisiae)	10.4	92.4
Il1rl1	NM_010743	interleukin 1 receptor-like 1, transcript variant 2	10.3	6.7
Serpinb2	NM_011111	serine (or cysteine) peptidase inhibitor, clade B, member 2	10.1	29.5
Bst1	NM_009763	bone marrow stromal cell antigen 1	9.2	8.8
Timp1	NM_011593	tissue inhibitor of metalloproteinase 1, transcript variant 2	9.2	8.4
Steap1	NM_027399	six transmembrane epithelial antigen of the prostate 1	7.4	6.1
Saa3	NM_011315	serum amyloid A 3	6.3	72.7
Rasgrf1	NM_001039655	RAS protein-specific guanine nucleotide-releasing factor 1	6.2	9.4
Slc15a3	NM_023044	solute carrier family 15, member 3	6.1	28.7
Clec4e	NM_019948	C-type lectin domain family 4, member e	6.1	7.4
Mmp3	NM_010809	matrix metallopeptidase 3	6.1	41.6
Lcn2	NM_008491	lipocalin 2	5.6	16.7

DNA microarray analysis was repeated with the Cy3 and Cy5 dyes reversed (a dye swap). Fold change (*Fold 1*) represents the average of mRNA expression level in *db*/*db* mice relative to *db*/+ mice. Fold change (*Fold 2*) represents the average of mRNA expression level in the 3T3-L1 adipocytes upon co-culture with macrophages in the presence of LPS relative to with macrophages in the absence of LPS.
