# Supplementary material for: Serum Amyloid A3 Gene Expression in Adipocytes is an Indicator of the Interaction with Macrophages
Source: Sci Rep. 2016 Dec 8;6:38697. doi: 10.1038/srep38697 (PMC5144138; doi:10.1038/srep38697)

Serum Amyloid A3 Gene Expression in Adipocytes is an Indicator of the Interaction with Macrophages

Yohei Sanada, Takafumi Yamamoto, Rika Satake, Akiko Yamashita, Sumire Kanai, Norihisa Kato, Fons AJ van de Loo, Fusanori Nishimura, Philipp E. Scherer and Noriyuki Yanaka

Supplementary Figures:

Supplementary Figure 1

Supplementary Figure 2

Supplementary Figure 3

Supplementary Figure 4

Supplementary Figure 5

Supplementary Figure 6

Supplementary Figure 7

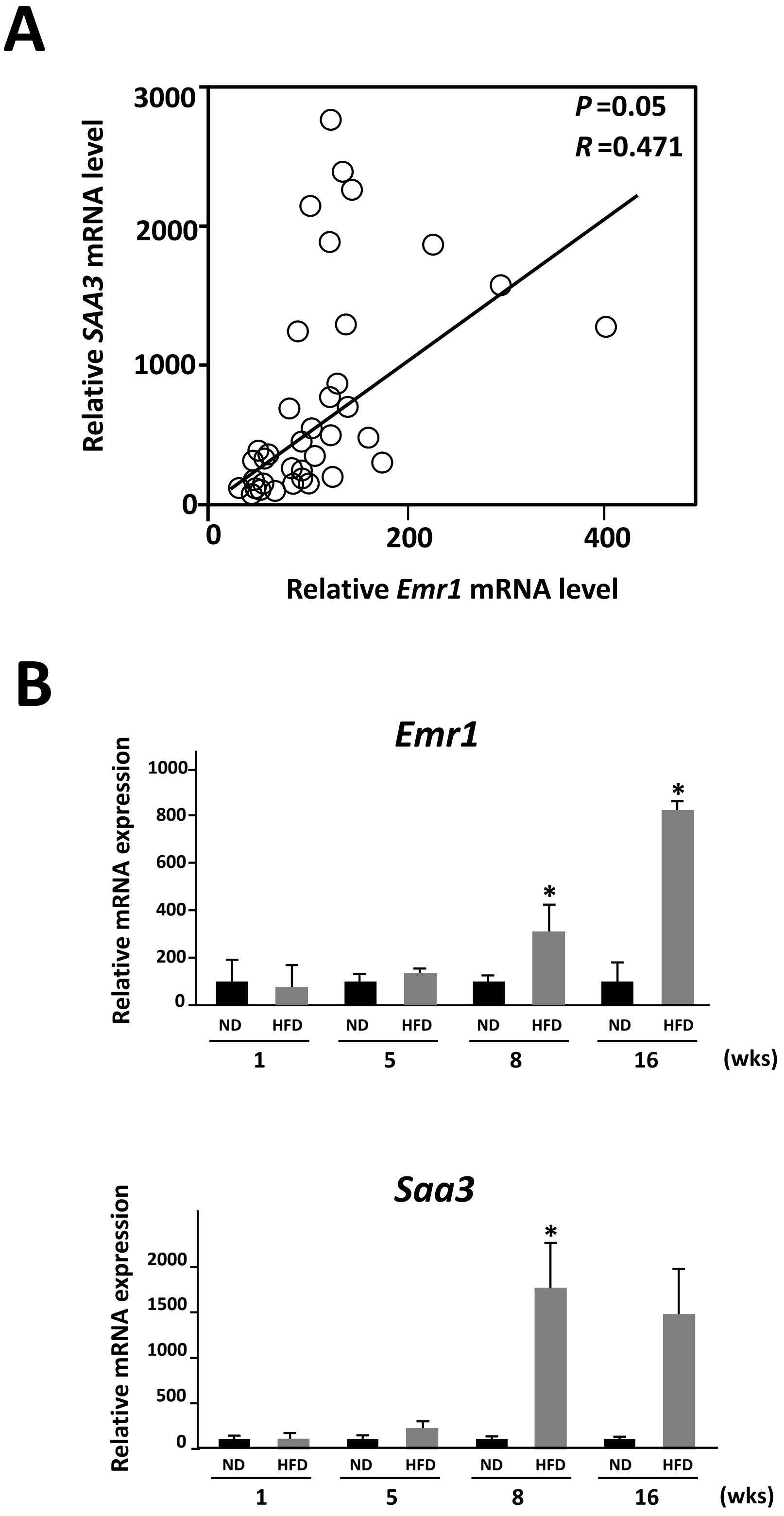

Supplementary Figure 1. Analysis of *Saa3* and *Emr1* mRNA expression in obese adipose tissue of HFD mice. (A) The relative mRNA expression level of *Saa3* and *Emr1* genes in the

epididymal white adipose tissue of mice fed HFD (n = 24) was determined by quantitative PCR and normalized to β-actin mRNA level. Pearson's correlation coefficient showed a positive correlation between Saa3 and *Emr1* mRNA levels in adipose tissue of HFD mice. (B) Total RNA from the epididymal adipose tissue of mice fed normal diet (*ND*) or high fat diet (*HFD*) for 1, 5, 8, 16 weeks (n=3) was subjected to quantitative PCR to examine mRNA expression level of *Saa3* and *Emr1* genes. Data represent mean ± SE. *$p<0.05$ compared with those of ND.

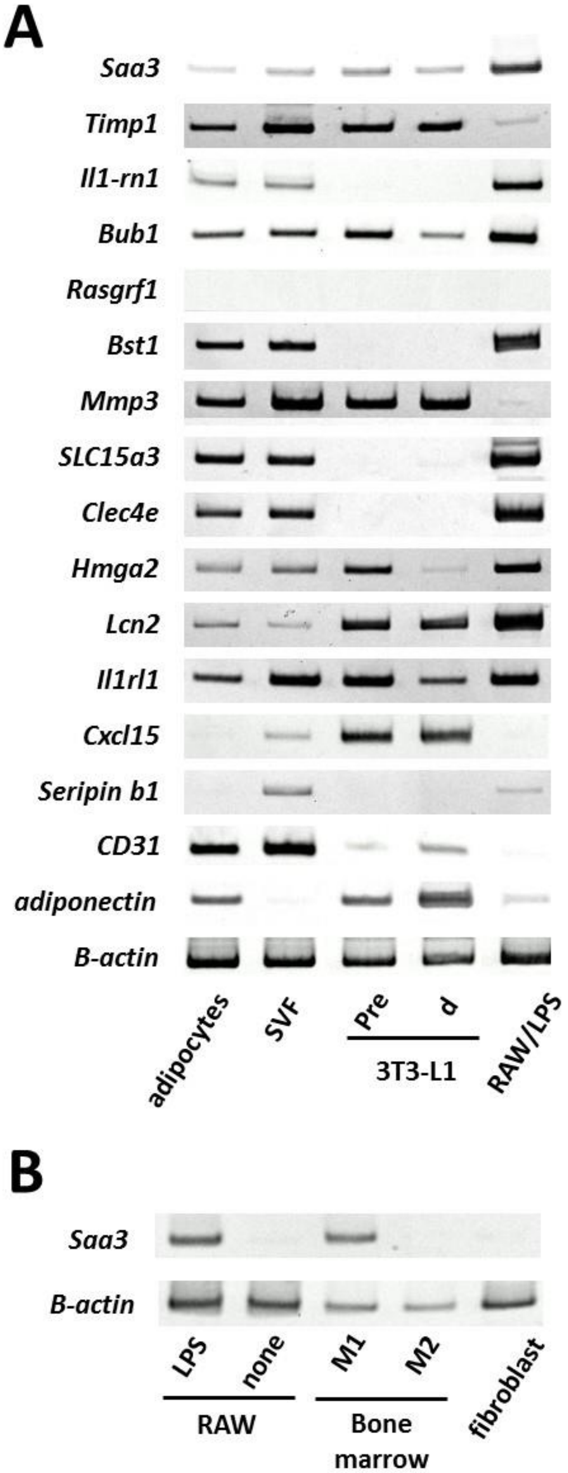

Supplementary Figure 2. mRNA expression profile of obesity-related genes in adipocytes and macrophages.

(A) Semiquantitative RT-PCR was performed to determine mRNA levels of 15 genes that were

found to be significantly up-regulated in obese adipose tissues. 3T3-L1 preadipocytes (*Pre*) were treated with MDI for 48 h and differentiated into mature adipocytes (*d*) as described under “Materials and Methods”. RAW264.7 cells were stimulated with 1 μg/ml of LPS for 18 hr (*RAW/LPS*). Mature adipocytes (*adipocytes*) and SVF were isolated from white adipose tissue of HFD mice as described under “Materials and Methods”. (B) Semiquantitative RT-PCR was performed to determine Saa3 mRNA level in RAW264.7 cells, M1 and M2 macrophages from bone marrow cells and adipose fibroblasts. Mouse bone marrow cells were suspended in macrophage medium (IMDM containing 20% FCS, 10 ng/ml recombinant human M-CSF, 2 mM L-glutamine, 50 U/ml penicillin, and 50 μg/ml streptomycin) and cultured in 5% CO2/95% humidified air for 6 days. Bone marrow-derived macrophage cells were cultured in macrophage medium without M-CSF for 48 h and further cultured in the presence of 10ng/ml mouse IL-4 or 20ng/ml IFN-γ plus 100ng/ml LPS for 24 h. To isolate adipose fibroblasts, epididymal white adipose tissue isolated from male HFD mice was minced in phosphate-buffered saline and digested with 1 mg/ml collagenase Type I (Worthington Chemical Corporation) for 30 min at 37°C. The resulting cell suspension was filtered through a 100-μm filter and centrifuged at 233 × g for 1 min. Isolated cells were cultured in a maintenance medium (10% fetal bovine serum, 100 units/ml penicillin and 100μg/ml streptomycin in DMEM) in 5% CO2/95% humidified air. After reaching confluence, cells were transferred to new dishes containing fresh maintenance medium. This step was repeated twice, and total RNA from adipose fibroblasts was subjected to RT-PCR analysis.

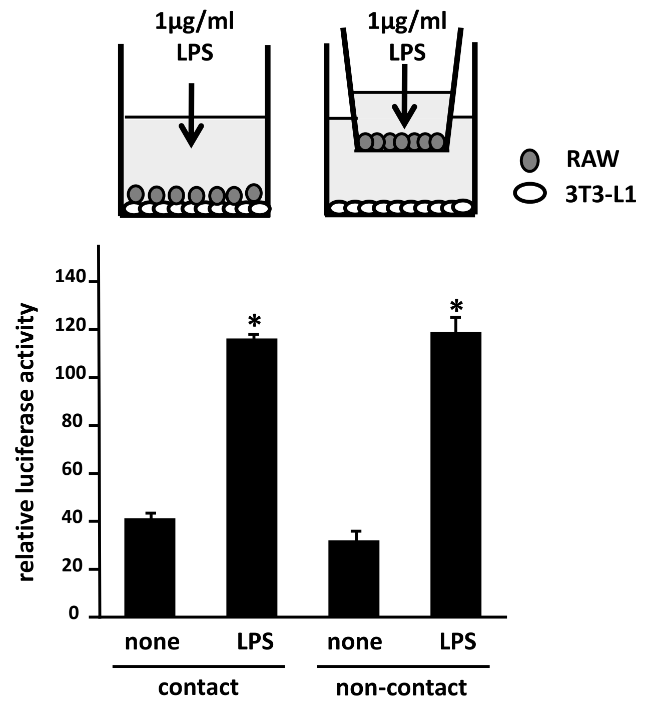

Supplementary Figure 3. Saa3 promoter activity in 3T3-L1 adipocytes is affected in the presence of RAW264.7 macrophages.

Illustration of the contact coculture system composed of 3T3-L1/Saa3-luc adipocytes and RAW264.7 macrophages. 1 x 10^5 3T3-L1 cells were treated with differentiation medium (maintenance medium plus 0.5mM 3-isobutyl-1-methylxanthine (IBMX), 5μg/ml insulin, and 1μM dexamethasone (DEX), MDI) and incubated for 2 days. Then, differentiation medium was replaced with growth medium (maintenance medium supplemented 5μg/ml insulin), which was refreshed every 2 days. 5 x 10^4 RAW264.7 cells were plated onto differentiated 3T3-L1 cells and co-cultured for 2 day, and thereafter, stimulated with 1 μg/ml of LPS for 24 hr. Luciferase activity in the 3T3-L1 adipocytes was examined upon co-culture with macrophages activated by LPS. * $p < 0.05$.

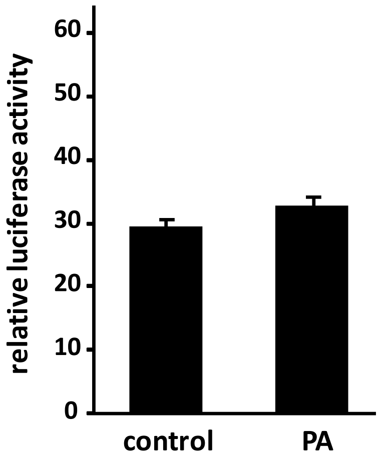

Supplementary Figure 4. Effect of palmitic acid on the saa3 promoter activity in 3T3-L1 adipocytes.

1 x 10^5 3T3-L1 cells were treated with differentiation medium (maintenance medium plus 0.5mM 3-isobutyl-1-methylxanthine (IBMX), 5µg/ml insulin, and 1µM dexamethasone (DEX), MDI) and incubated for 2 days. Then, differentiation medium was replaced with growth medium (maintenance medium supplemented 5µg/ml insulin), which was refreshed every 2 days. Palmitic acid (PA) was dissolved in 100% ethanol and a 10mM PA stock solution was prepared in 1N NaOH by heating at 60◦C in shaking water bath. After saponification reaction, PA stock solution was evaporated at 55◦C for 90 min in evaporation rotor. After dryness, PA was dissolved in 0.9% (wt/vol) NaCl solution. 10% (wt/vol) free fatty acid-free bovine serum albumin (BSA) solution was prepared in 0.9% NaCl solution. 5 mM PA/5% BSA stock solution was prepared by adding 4 ml of the 10 mM PA solution dropwise to 4 ml 10% BSA solution at 45◦C in a shaking water bath, then vortex mixed for 10 sec followed by a further 10 min incubation at 55◦C. The PA/BSA complexed solution is cooled to room temperature and diluted with DMEM solution to 500 µM, then sterile filtered (0,45 µm pore size membrane filter). 3T3-L1 cells were stimulated with 500 µM of PA for 24 hr. Luciferase activity in the 3T3-L1 adipocytes was examined.

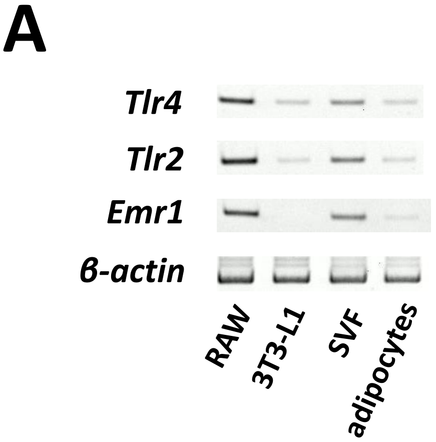

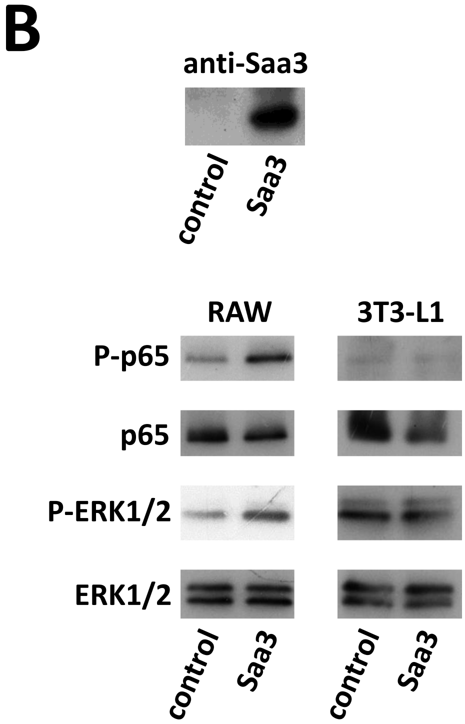

Supplementary Figure 5. Effect of Saa3 protein on adipocyte functions.

(A) Semiquantitative RT-PCR was performed to determine mRNA levels of *Tlr2* and *Tlr4* genes. RAW264.7 cells were stimulated with 1 μg/ml of LPS for 18 hr (*RAW*). 3T3-L1 preadipocytes were differentiated into mature adipocytes (*3T3-L1*) as described under "Materials and Methods". Mature adipocytes (*adipocytes*) and SVF were isolated from white adipose tissue

of HFD mice as described under “Materials and Methods”. (B) HEK293T cells were transiently transfected with mouse Saa3 cDNA. Total protein extracts (10 μg/lane) from transfected HEK293T cells were subjected to SDS-PAGE followed by Western blotting using an anti-Saa3 antibody. RAW264.7 cells (*RAW*) and differentiated 3T3-L1 adipocytes (*3T3-L1*) were treated with conditioned medium of HEK293T cells for 15 min. Total cell lysates were prepared with RIPA buffer (50 mm Tris-HCl, pH 7.5, 2 mm EDTA, 0.5% deoxycholate, 150 mm NaCl, 1% Triton X-100, 0.1% SDS, 20 mm β-glycerophosphate, 1 mm sodium orthovanadate, 1 mm phenylmethylsulfonyl fluoride, 10 μg/ml aprotinin, and 10 μg/ml leupeptin). Total protein extracts (10 μg/lane) were separated SDS–PAGE using a 10% polyacrylamide gel, transferred to Immobilon P filters (Millipore, Bedford, MA) and subjected to Western blot analyses using antibody specific for phosphorylated form of p65 (upper panel) or ERK1/2 (lower panel). The same membrane was re-blotted with anti-p65 antibody or anti-ERK1/2 antibody. The anti-phospho-NF-κB p65 and anti-phospho-ERK1/2 antibodies were purchased from Cell Signaling Technology (Beverly, MA). Antibodies against NF-κB p65, ERK1 and ERK2 were obtained from Santa Cruz Biotechnologies Inc. (Santa Cruz, CA).

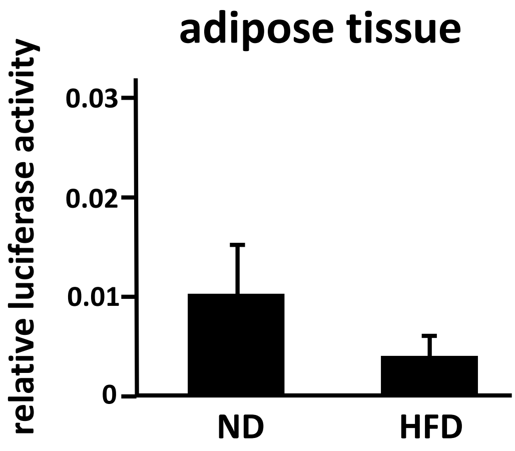

Supplementary Figure 6. Saa3 promoter activity in transgenic mice fed HFD.

Transgenic mice carrying Saa3-luc (Saa3-luc mouse) were fed either ND or HFD for 1 week. The epididymal white adipose tissue of Saa3-luc mice were isolated and subjected to the luciferase activity. The data (mean ± S.E.) are from a single experiment carried out (n = 3) and are representative of two independent experiments.

Supplementary Figure 7

Fig. 5F

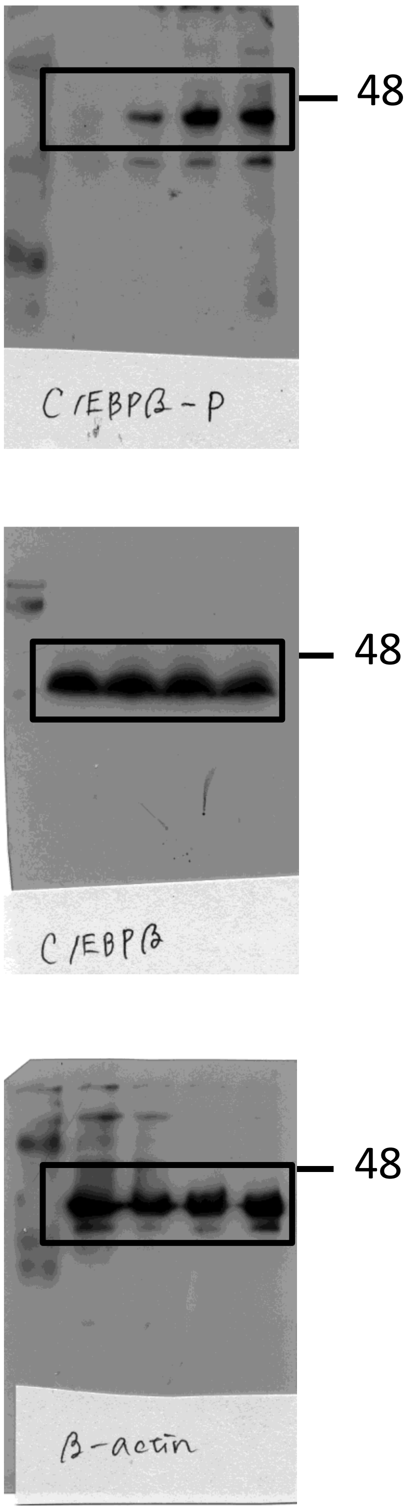

Supplement: Supplementary Information [file srep38697-s1.pdf]
